# Depth estimation from wavelength-dependent fluorescence spread profiles across NIR-I and NIR-II

**DOI:** 10.1117/1.JBO.31.9.096004

**Published:** 2026-09-16

**Authors:** Dogancan Kuyel, Zhongmin Zhu, Brianna Hajek, Sinisha Stojanovski, Anamarija Jankulovska, Igorce Djikovski, Risto Colanceski, Imran Ferati, Natasha Toleska Dimitrovska, Ilir Vela, Despot Despotovski, Nexhati Jakupi, Darko Angjushev, Magdalena Bogdanovska Todorovska, Goran Kondov, Borislav Kondov, Fumihiro Kawano, Shuming Nie, Claudius Conrad, Viktor Gruev

**Affiliations:** a University of Illinois at Urbana-Champaign, Department of Bioengineering, Urbana, Illinois, United States; b University of Illinois at Urbana-Champaign, Department of Electrical and Computer Engineering, Urbana, Illinois, United States; c Ss. Cyril and Methodius University of Skopje, Institute of Pathophysiology and Nuclear Medicine, Skopje, Republic of North Macedonia; d Ss. Cyril and Methodius University of Skopje, Department of Thoracic and Vascular Surgery, Skopje, Republic of North Macedonia; e Ss. Cyril and Methodius University of Skopje, Institute of Pathology, Skopje, Republic of North Macedonia; f Carle Illinois College of Medicine, Urbana, Illinois, United States; g University of Illinois at Urbana-Champaign, Beckman Institute for Advanced Science and Technology, Urbana, Illinois, United States

**Keywords:** fluorescence depth estimation, multispectral imaging, indocyanine green, NIR-I, NIR-II, fluorescence-guided surgery

## Abstract

**Significance:**

Wide-field fluorescence-guided imaging is inherently depth ambiguous. Multispectral imaging across NIR-I and NIR-II may encode depth because longer-wavelength fluorescence undergoes less diffuse broadening in tissue.

**Aim:**

The study aims to determine whether wavelength-dependent radial spread profiles of subsurface indocyanine green (ICG) fluorescence can be used to estimate depth from a single excitation wavelength.

**Approach:**

ICG phantoms (1 to 50  μM) were imaged through *ex vivo* chicken breast under 785 nm excitation using four emission bands spanning NIR-I and NIR-II. Radial spread profiles from high-dynamic-range images formed a multispectral dictionary, and depth was estimated with an inverse model evaluated leave-one-thickness-out across 2.2 to 6.0 mm under concentration-matched and concentration-randomized conditions, randomized exposure subsets, and spectral-band ablations, and compared with analytical and empirically calibrated scalar dual-emission fluorescence-ratio baselines of Leblond et al. Two ICG-localized sentinel lymph nodes from a breast cancer specimen were analyzed *ex vivo*.

**Results:**

Longer-wavelength channels produced more compact spread profiles, and full width at 20% peak intensity increased linearly with tissue thickness for all filters ($R^{2}=0.94$ to 0.95). The NIR-II-only subset (940 and 1070 nm) yielded sub-millimeter root mean square error at every held-out thickness under both concentration conditions (0.32 to 0.66 mm), whereas the analytical and empirical scalar-ratio baselines yielded 1.58 to 5.24 and 1.13 to 2.74 mm. Clinical sentinel lymph nodes preserved the wavelength-dependent profile narrowing, with phantom-to-clinical normalized-width RMSE of 3.3% to 8.5%.

**Conclusions:**

Wavelength-dependent fluorescence spread profiles across NIR-I and NIR-II can encode depth information for subsurface ICG targets, providing a wide-field, single-fluorophore, single-excitation approach compatible with emerging multispectral surgical imaging platforms.

## 1 Introduction

Near-infrared fluorescence-guided surgery (FGS) has become an established intraoperative imaging adjunct across a growing range of procedures. Indocyanine green (ICG) remains the most widely used clinical fluorophore, while newer targeted agents such as pafolacianine (Cytalux) extend fluorescence guidance toward molecularly specific tumor visualization. Commercial platforms now support open, minimally invasive, robotic, and microscope-based workflows, yet most wide-field fluorescence systems still provide fundamentally planar images. Consequently, the measured fluorescence signal depends not only on fluorophore presence and concentration but also on tissue absorption and scattering, imaging geometry, and the depth of the fluorescent source beneath the tissue surface.^1^^,^^2^

Recovering source depth from planar fluorescence images remains challenging. Tomographic, structured-light, and time-resolved techniques can provide depth information, but they generally require more complex instrumentation, acquisition strategies, and reconstruction pipelines than are practical for many real-time surgical workflows.^3^

A substantial body of prior work has therefore sought simpler depth-sensitive observables derived from multispectral fluorescence measurements. Swartling et al. first showed that wavelength-dependent spectral deformation of fluorescence propagating through turbid tissue can encode lesion depth. Leblond et al. later derived a diffusion-based analytical model relating the logarithm of the fluorescence ratio at two emission wavelengths to source depth under homogeneous, diffuse, and point-source assumptions. This framework was translated to wide-field imaging by Kolste et al., who demonstrated fluorescence topography, and by Jermyn et al., who used ratio-derived depth maps as priors for subsequent subsurface quantitative reconstruction. Related studies have also explored dual-fluorophore and dual-excitation strategies, as well as alternative nontomographic depth cues such as dual-aperture fluorescence ratio imaging. More recently, García et al. emphasized that the accuracy of fluorescence ratio-based depth estimation depends strongly on how well the forward model matches the true measurement geometry and optical boundary conditions.^4^^–^^12^

Recent advances in multispectral and shortwave-infrared (SWIR/NIR-II) imaging are especially relevant to this problem. Multispectral systems can separate multiple fluorophores or interrogate the same fluorophore across several detection bands, and SWIR/NIR-II imaging is emerging as an important modality in biomedical optics. Because tissue scattering arises from a combination of Rayleigh-like and Mie-like processes, the reduced scattering coefficient generally decreases with increasing wavelength and is commonly approximated by a power law, $\mu_{s}^{\prime }(\lambda )\propto \lambda^{-\alpha }$. As a result, longer-wavelength fluorescence undergoes less diffuse broadening than shorter-wavelength fluorescence and can yield sharper images with reduced background from scattering and autofluorescence. These advantages have been highlighted in recent SWIR/NIR-II reviews and experimental demonstrations, and ICG itself exhibits a measurable long-wavelength emission tail that can be detected beyond conventional NIR-I.^2^^,^^13^^–^^17^

**Fig. 1:**
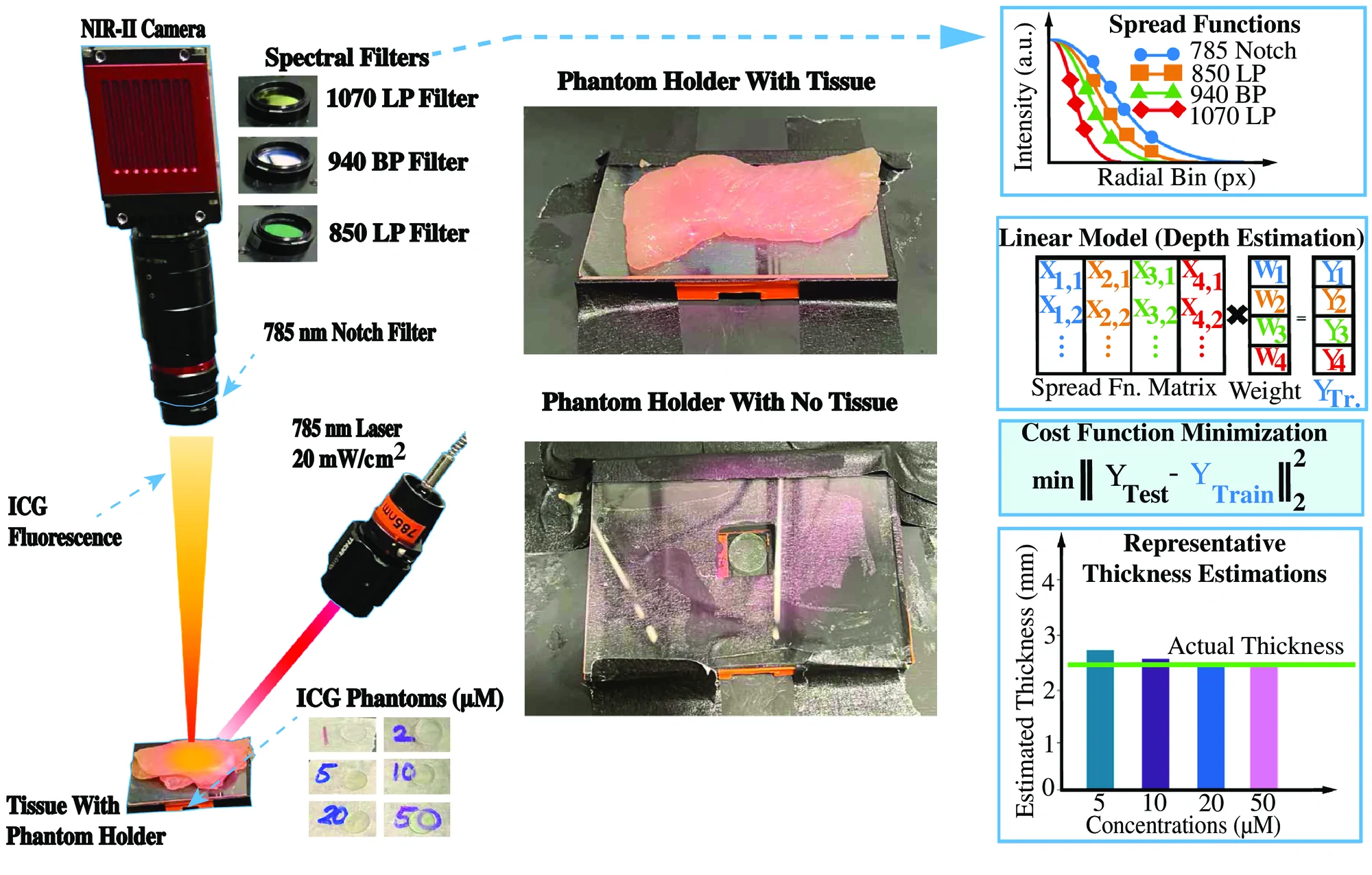
Overview of the proposed multispectral fluorescence depth-estimation approach. ICG-doped phantoms are excited with a 785 nm laser and imaged with a SWIR camera using spectral filters spanning NIR-I and NIR-II. A fixed tissue–phantom interface enables controlled measurements across different tissue thicknesses. Multispectral images are converted into wavelength-dependent radial spread profiles, where longer-wavelength emission produces a more compact apparent blur than shorter-wavelength emission. Profiles from known thicknesses are used to build the training matrix, and an unknown profile is compared against this dictionary in an alternating direction method of multipliers (ADMM)-based inverse model to estimate tissue depth.

In this work, we leverage a broadband imaging platform with detector sensitivity from 400 to 1700 nm to interrogate the same ICG source across multiple emission bands spanning conventional NIR-I and extending toward the NIR-II/SWIR transition regime (Fig. 1). We hypothesize that, for a buried ICG source excited at 785 nm, the apparent fluorescence profile at the tissue surface becomes progressively more compact at longer detection wavelengths because tissue scattering decreases with wavelength. Rather than relying solely on scalar intensity ratios, we use the wavelength dependence of the full spatial spread profile, or fluorescence blur profile, as the depth-bearing observable. Multispectral spread profiles extracted from a single fluorophore under one excitation wavelength are then assembled into an inverse model to estimate source depth while remaining compatible with wide-field fluorescence-guided imaging workflows. An overview of the imaging platform, the wavelength-dependent spread-profile concept, and the reconstruction workflow is shown in Fig. 1.^18^

## 2 Methods

### 2.1 Imaging System and Sample Configuration

Fluorescence imaging was performed with a visible-SWIR camera (Allied Vision Goldeye G-130 SWIR TEC1, Taschenweg, Stadtroda, Germany) mounted above the sample plane and fitted with a Kowa VIS-SWIR lens (Kowa LM16HC-VIS-SW, 16 mm focal length, 1.8 to 16 Fstop, Nagoya, Aichi, Japan). Excitation was provided by a 785 nm laser diode (Innovative Photonic Solutions, I0785MM6000 MS-7UP) adjusted to an incident irradiance of $∼20\;\mathrm{mW}/{\mathrm{cm}}^{2}$ at the sample surface. Emission was sampled sequentially with a 785 nm notch filter (Chroma Technology Corp., ZET785TopNotch, Vermont, United States), an 850 nm long-pass filter (Midwest Optical Systems, LP850-25.4), a 940 nm band-pass filter (Midwest Optical Systems, Bi940-25.4, FWHM 32 nm, Palatine, United States), and a 1070 nm long-pass filter (Midwest Optical Systems, LP1070-25.4). The camera-to-sample distance was 24 cm, and the field of view was 8.5×11  cm at the sample plane. ICG emission in tissue-like media peaks in the NIR-I window near 810 to 820 nm and decreases monotonically with increasing wavelength, retaining a measurable emission tail well beyond 1000 nm that is orders of magnitude weaker than the peak. The four detection bands sample this curve at progressively longer wavelengths. The 785 nm notch filter rejects the excitation line while passing emission on both sides of it, placing this channel nearest the emission maximum; the 850 nm long-pass channel integrates the remaining NIR-I emission; and the 940 nm band-pass and 1070 nm long-pass channels sample the NIR-II/SWIR emission tail reported by Carr et al. Because tissue scattering decreases with wavelength while ICG emission also decreases, these filters were selected to span the range over which wavelength-dependent radial spread-profile differences remain detectable, enabling us to test whether they improve dictionary-based depth estimation of ICG targets under a single 785 nm excitation.^17^

ICG-doped phantoms were prepared at concentrations of 1, 2, 5, 10, 20, and 50  μM using clear V5 resin (Formlabs) and Indocyanine Green (TCI AMERICA). Phantoms were inserted into a custom sliding holder made up of PLA with dimensions of 5.2×7.8×0.8  cm that preserved the lateral position of the fluorescent target within the camera field of view during exchange between samples. *Ex vivo* chicken breast was used as the overlying scattering tissue. Tissue slabs of nominal thickness 2.2, 2.5, 4.0, 6.0, and 8.5 mm were placed above the holder. A transparent glass slide with dimensions of 5.1  cm×7.5  cm×0.1  mm (FisherBrand) maintained a reproducible tissue–phantom interface and minimized positional changes during repeated acquisitions.

### 2.2 Image Acquisition and Preprocessing

For each combination of tissue thickness, ICG concentration, and spectral filter, images were acquired at nine unique exposure times from 1 to 5000 ms together with a matched dark frame. When raw files were stored as short temporal stacks, 10 frames were averaged at each exposure prior to further processing. Dark-subtracted multiexposure images were fused into a single high-dynamic-range (HDR) image for each spectral band using a saturation-aware weighted average; the HDR formulation is given in Appendix A.

To evaluate robustness to acquisition conditions, the reconstruction analysis was repeated using four exposure configurations. The baseline HDR condition used the complete nine-exposure stack for each spectral band and was computed once. For the randomized exposure-subset conditions, a seed-specific exposure-selection manifest was generated by processing each acquisition group independently, where a group was defined as all images with the same thickness, concentration, and spectral filter, differing only in exposure time. Within each group, all available exposure times were collected and divided into low-exposure candidates satisfying $1\;\mathrm{ms}<t_{e}\leq 50\;\mathrm{ms}$ and high-exposure candidates satisfying $t_{e}\geq 200\;\mathrm{ms}$; one exposure was then randomly selected from each candidate list. The low-exposure condition used only the selected low exposure, whereas the high-exposure condition used only the selected high exposure. The low and high exposure condition fused the same selected low- and high-exposure images from the corresponding seed-specific manifest, rather than drawing an additional independent exposure pair. This procedure was repeated using three random seeds, producing three distinct randomized low/high exposure-selection patterns across the dataset, and predictions from the randomized exposure-subset conditions were averaged across seeds. All other preprocessing, profile extraction, dictionary construction, and inverse-model parameters were kept fixed across exposure configurations.

### 2.3 Spread-Profile Extraction

For each spectral band v, a rectangular region of interest (ROI) containing the fluorescent target was selected from the HDR image $I_{v}(x,y)$. The target center $c=(c_{x},c_{y})$ was refined from the local intensity distribution within the ROI, and the fluorescence spread profile was then computed by averaging image intensity over integer-radius annuli centered at c. The ring-mean profile was defined as

$$p_{v}(k)=\frac{1}{\left|R_{k}\right|}\underset{(x,y)\in R_{k}}{\sum }I_{v}(x,y),\;R_{k}=\{(x,y):k\leq r(x,y;c)<k+1\},$$ (1)

where r(x,y;c) is the Euclidean distance from (x,y) to the refined centroid and $\left|R_{k}\right|$ is the number of pixels in annulus $R_{k}$.

For visualization, profiles were peak-normalized. For reconstruction, the nonnegative postpeak portion of each profile was area-normalized,

$$y_{v}(k)=\frac{p_{v}(k)}{\underset{k}{\sum }p_{v}(k)},$$ (2)

so that the inverse model operated on profile shape rather than absolute intensity.

### 2.4 Multispectral Dictionary Construction

For each known tissue thickness $d_{i}$, the normalized spread profiles from all V spectral bands were concatenated to form a multispectral dictionary atom,

$$x_{i}=[\begin{array}{c} x_{1,i}\\ x_{2,i}\\ \vdots \\ x_{V,i} \end{array}],\;y=[\begin{array}{c} y_{1}\\ y_{2}\\ \vdots \\ y_{V} \end{array}],\;X=[x_{1}x_{2}\cdots x_{N}],$$ (3)

where N is the number of training thicknesses and y is the stacked unknown test profile. Each entry of the depth-weight vector $a\in R^{N}$ describes the contribution of thickness $d_{i}$ to the measured multispectral spread profile.

### 2.5 Dictionary-Based Depth Reconstruction

Depth estimation was formulated as a weighted inverse problem. Let W denote a diagonal weighting matrix that down-weights less reliable spectral bands and up-weights bands with stronger depth sensitivity. The depth-weight vector a was estimated by solving

a^=arg mina 12‖W(y−Xa)‖22+λTV‖∇a‖1subject to  a≥0,  1Ta=1, (4)

where ∇ is the first-difference operator across depth and $\lambda_{\mathrm{TV}}$ controls the total-variation penalty. The simplex constraint ($a\geq 0,1^{T}a=1$) forces the recovered depth weights to behave like a probability distribution over the available thicknesses, while the total-variation term discourages fragmented solutions across nonadjacent depths.

Equation (4) was solved using the alternating direction method of multipliers (ADMM). In the current implementation, W combines fixed spectral weights with an adaptive robust reweighting based on the residuals between measured and reconstructed spread profiles. The code path currently also applies a small per-band amplitude correction and a local per-band dilation correction to reduce residual mismatch between training and test profiles. The corresponding update equations are collected in Appendix B.

For each test acquisition, the residual vector was computed as the bin-wise difference between the measured multispectral spread profile y and the dictionary reconstruction Xa, with its entries spanning all radial bins and spectral bands. The solver converted these weighted residuals into a single scalar cost, $\frac{1}{2}\|W(y-Xa){\|}_{2}^{2}$, and iteratively updated the depth-weight vector a to minimize this cost together with the total-variation regularization term. Consequently, the method did not assign depth on a per-pixel basis; instead, it produced one depth-weight distribution and, therefore, one scalar depth estimate for each unknown ROI-derived spread profile. The final depth estimate was computed from the recovered depth-weight distribution as

d^=∑i=1Naidi. (5)

### 2.6 Evaluation Protocol

For each ICG concentration, multispectral spread profiles acquired at known tissue thicknesses were used to construct the dictionary in Eq. (3). Depth-reconstruction performance was evaluated using a leave-one-thickness-out protocol. Five independently cut chicken-breast tissue slabs were used to generate the five nominal thickness conditions, with slab thickness measured by calipers. Although data were acquired at five nominal tissue thicknesses, 2.2, 2.5, 4.0, 6.0, and 8.5 mm, the 8.5 mm condition was excluded from the evaluable test set because the fluorescence signal approached the camera/SNR limit, particularly in the longer-wavelength channels. The evaluable test set was therefore D={2.2,2.5,4.0,6.0} mm. For each held-out test thickness $d_{\text{test}}\in D$, all profiles acquired at $d_{\text{test}}$ were excluded from training, and the dictionary was constructed only from profiles acquired at the remaining three evaluable thicknesses together with the 8.5 mm condition. Thus, the 8.5 mm profiles were retained as training-only dictionary entries in every leave-one-thickness-out fold, but the 8.5 mm condition was not evaluated as a held-out test thickness or included in the reported reconstruction-error summaries. No profile acquired at the held-out thickness contributed to the corresponding training dictionary. Furthermore, depth estimation was performed under concentration-matched and concentration-randomized conditions. In the concentration-matched setting, held-out test profiles were reconstructed using a dictionary of spread profiles with known depth labels acquired at the same ICG concentration. In the concentration-randomized setting, held-out test profiles were reconstructed using a dictionary of spread profiles with known depth labels acquired at different ICG concentrations to assess robustness to unknown fluorophore concentration across the 5 to 50  μM range. The predicted depth was computed from Eq. (5), and prediction error was defined as

ed=d^−dtrue. (6)

For the summary plots, reconstruction accuracy was reported as the root mean square error (RMSE), with error bars denoting the standard error of the mean (SEM) over the corresponding averaging dimension. In Fig. 5(a), concentration prediction error for each concentration was summarized by averaging RMSE across all test thicknesses, with SEM computed across thicknesses. In Fig. 5(b), thickness prediction error for each thickness was summarized by averaging RMSE across concentration test conditions, including both concentration-matched and concentration-randomized cases separately, with SEM computed across those concentration conditions.

### 2.7 Radial Spread Profile Correlation with Tissue Thickness

To quantify the relationship between radial profile width and overlying tissue thickness, we computed a scalar full width at 20% of the peak intensity value (P20) from each peak-normalized radial spread profile. For each spectral band v, concentration c, and tissue thickness d, the annular profile $p_{v,c,d}(k)$ was first peak normalized as

$$q_{v,c,d}(k)=\frac{p_{v,c,d}(k)}{\underset{j}{\max}\;p_{v,c,d}(j)}.$$ (7)

Let $k_{\text{peak}}$ denote the radial bin of the profile maximum. Starting after the peak, the first pair of adjacent radial bins satisfying

$$q_{v,c,d}(k_{i}-1)\geq 0.20,\;q_{v,c,d}(k_{i})\leq 0.20$$ (8)

was identified. The 20% crossing radius was then obtained by linear interpolation:

$$r_{20}=r_{i-1}+\frac{0.20-q_{v,c,d}(k_{i}-1)}{q_{v,c,d}(k_{i})-q_{v,c,d}(k_{i}-1)}\;(r_{i}-r_{i-1}).$$ (9)

The full P20 width was defined as

$$w_{20}=2r_{20}.$$ (10)

Profiles that did not cross the 20% peak-intensity level within the measured radial window were excluded from the width-correlation analysis rather than extrapolated. Measurements were pooled across ICG concentrations within each spectral filter and included the zero-thickness (no-tissue) condition, giving n=24 measurements for the 850 nm long-pass, 940 nm band-pass, and 1070 nm long-pass filters and n=22 for the 785 nm notch filter, for which two profiles did not cross the 20% level. The zero-thickness condition is included in this width analysis only; it was not used as a dictionary entry in the depth-reconstruction model.

For each spectral filter, P20 width was modeled as a linear function of tissue thickness:

$$w_{20,i}=\beta_{0,v}+\beta_{1,v}d_{i}+\epsilon_{i},$$ (11)

where $w_{20,i}$ is the measured P20 width for measurement i, $d_{i}$ is the corresponding tissue thickness, v indexes the spectral filter, $\beta_{0,v}$ is the filter-specific intercept, $\beta_{1,v}$ is the filter-specific slope, and $\epsilon_{i}$ is the residual error between the measured width and the fitted line. Ordinary least-squares regression was used to estimate β^0,v and ${\hat{\beta }}_{1,v}$ by minimizing the sum of squared residuals:

(β^0,v,β^1,v)=arg minβ0,v,β1,v∑i[w20,i−(β0,v+β1,vdi)]2. (12)

After fitting the regression line, the predicted P20 width for each measurement was computed as

w^20,i=β^0,v+β^1,vdi. (13)

Here, $w_{20,i}$ denotes the experimentally measured P20 width, whereas w^20,i denotes the fitted or predicted P20 width from the linear regression model. The mean measured P20 width for each spectral filter was defined as

$${\overset{¯}{w}}_{20,v}=\frac{1}{n_{v}}\overset{n_{v}}{\underset{i=1}{\sum }}w_{20,i},$$ (14)

where $n_{v}$ is the number of valid P20-width measurements for filter v. The coefficient of determination was then computed as

Rv2=1−∑i(w20,i−w^20,i)2∑i(w20,i−w¯20,v)2. (15)

In this expression, the numerator represents the residual variation remaining after fitting the linear model, while the denominator represents the total variation in measured P20 width before fitting. Therefore, $R_{v}^{2}$ quantifies the fraction of P20-width variation explained by tissue thickness for each spectral filter.

### 2.8 Analytical and Empirical Scalar-Ratio Baseline Analyses

Both scalar-ratio baselines were evaluated using the 785 nm notch and 850 nm long-pass channels, with the ratio oriented as 785 nm over 850 nm. Let $S_{\lambda ,i}(p)$ denote the temporally averaged and matched dark-subtracted signal at pixel p for measurement i and spectral channel λ. Specifically,

$$S_{\lambda ,i}(p)=\frac{1}{N_{\lambda ,i}}\overset{N_{\lambda ,i}}{\underset{k=1}{\sum }}I_{\lambda ,i,k}(p)-\frac{1}{N_{\lambda ,i}^{\text{dark}}}\overset{N_{\lambda ,i}^{\text{dark}}}{\underset{k=1}{\sum }}I_{\lambda ,i,k}^{\text{dark}}(p),$$ (16)

where $I_{\lambda ,i,k}$ and $I_{\lambda ,i,k}^{\text{dark}}$ are the signal and matched-dark frames, respectively. When temporal image stacks were available, 10 frames were averaged before the ratio calculation. A common 81×78-pixel region of interest (ROI), denoted by $\Omega_{i}$, was used for both baseline methods. Logarithms and depth estimates were evaluated only over the valid pixel set $\Omega_{i}^{+}\subseteq \Omega_{i}$, for which the dark-subtracted signal was positive in both channels.

Unlike the multispectral spread-profile analysis, the scalar-ratio baselines were applied to individual-exposure images rather than HDR reconstructions. The 785 and 850 nm images within each tissue measurement used a common exposure time. The corresponding 785 and 850 nm zero-thickness calibration images also used a common exposure time, although a shorter exposure and low camera gain were used for these bright calibration measurements to avoid detector saturation. For each concentration, the zero-thickness calibration images were acquired from the same ICG phantom used in the corresponding tissue measurements, thereby providing a preparation-matched calibration reference.

#### 2.8.1 Analytical scalar-ratio baseline

The analytical baseline followed the diffusion-based fluorescence-ratio relationship originally derived by Leblond et al.^5^ and subsequently evaluated by García et al.^12^ For fluorescence detected at two wavelengths, $\lambda_{1}$ and $\lambda_{2}$, the analytical model predicts

$$\ln\;\Gamma^{\infty }(d)=-(\frac{1}{\delta_{\lambda_{1}}}-\frac{1}{\delta_{\lambda_{2}}})d+\ln(\frac{D_{\lambda_{2}}}{D_{\lambda_{1}}}),$$ (17)

where $\Gamma^{\infty }(d)=I_{\lambda_{1}}(d)/I_{\lambda_{2}}(d)$ is the two-wavelength fluorescence ratio predicted under the homogeneous diffusion-model assumptions, d is source depth, and $D_{\lambda }$ is the wavelength-dependent diffusion coefficient,

$$D_{\lambda }=\frac{1}{3(\mu_{a,\lambda }+\mu_{s,\lambda }^{\prime })},$$ (18)

with $\mu_{a,\lambda }$ denoting the absorption coefficient and $\mu_{s,\lambda }^{\prime }$ denoting the reduced scattering coefficient. The corresponding optical penetration-depth parameter is

$$\delta_{\lambda }=\sqrt{\frac{D_{\lambda }}{\mu_{a,\lambda }}}.$$ (19)

Published *ex vivo* chicken-breast optical properties were used because sample-specific absorption and reduced-scattering coefficients were not independently measured. For the 785 nm notch channel, the closest available tabulated chicken-breast values were the 751 nm measurements reported by Kienle et al.,^19^ for which $\mu_{a,751}=0.0027\;{\mathrm{mm}}^{-1}$ and $\mu_{s,751}^{\prime }=0.28\;{\mathrm{mm}}^{-1}$. For the 850 nm long-pass channel, the unexposed chicken-breast measurements reported by Raymond et al.^20^ were used, for which $\mu_{a,850}=0.039\;{\mathrm{mm}}^{-1}$ and $\mu_{s,850}^{\prime }=0.392\;{\mathrm{mm}}^{-1}$. These coefficients give $D_{751}=1.1791\;\mathrm{mm}$, $\delta_{751}=20.8975\;\mathrm{mm}$, $D_{850}=0.7734\;\mathrm{mm}$, and $\delta_{850}=4.4532\;\mathrm{mm}$. Therefore, for the measured 785-over-850 ratio, the analytical slope and intercept are

$$m_{\mathrm{ana}}=-(\frac{1}{\delta_{751}}-\frac{1}{\delta_{850}})=\frac{1}{\delta_{850}}-\frac{1}{\delta_{751}}=0.176707\;{\mathrm{mm}}^{-1},b_{\mathrm{phys}}=\ln(\frac{D_{850}}{D_{751}})=-0.421722.$$ (20)

The 751 and 850 nm values should be interpreted as the available published wavelength proxies for the selected filters, rather than as sample-specific or passband-integrated optical properties. In particular, the 751 nm coefficients approximate the 785 nm notch channel, whereas the point values at 850 nm approximate the broad 850 nm long-pass measurement.

##### 2.8.1.1 Relative low- and high-gain characterization

Unlike García et al., who acquired the zero-thickness calibration and tissue images under consistent camera settings, we used different acquisition settings to accommodate the substantially brighter zero-thickness signal. Specifically, the tissue images were acquired at 10 ms, whereas the zero-thickness calibration images were acquired at 3 ms using the camera’s low-gain mode to avoid detector saturation. For each acquisition type, the 785 and 850 nm images were collected using the same exposure time. The effect of the gain-setting difference on the relative spectral response was evaluated independently using the wavelength-specific exposure-response analysis described below.

For each wavelength λ∈{785,850}, gain mode g∈{L,H}, and exposure time t, a dark-subtracted ROI statistic was calculated as

$$M_{\lambda ,g}(t)=\underset{p\in \Omega_{\text{gain}}}{\text{median}}[{\overset{¯}{I}}_{\lambda ,g,t}(p)-{\overset{¯}{I}}_{\lambda ,g,t}^{\text{dark}}(p)],$$ (21)

where only unsaturated measurements within the approximately linear detector-response range were included. A separate linear exposure response was then fitted for each wavelength and gain mode:

$$M_{785,L}(t)=m_{785,L}t+b_{785,L},\;M_{785,H}(t)=m_{785,H}t+b_{785,H},\;M_{850,L}(t)=m_{850,L}t+b_{850,L},\;M_{850,H}(t)=m_{850,H}t+b_{850,H}.$$ (22)

The channel-specific high-to-low gain factors were calculated from the fitted slopes,

$$G_{785}=\frac{m_{785,H}}{m_{785,L}},\;G_{850}=\frac{m_{850,H}}{m_{850,L}},$$ (23)

and the relative spectral gain factor was defined as

$$K_{\text{gain}}=\frac{G_{785}}{G_{850}}=\frac{m_{785,H}/m_{785,L}}{m_{850,H}/m_{850,L}}.$$ (24)

The fitted slopes, rather than the fitted intercepts, were used because camera gain is a multiplicative response, whereas the intercepts primarily reflect residual additive offsets and extrapolation to zero exposure. If the gain change were perfectly wavelength independent, then $G_{785}=G_{850}$ and $K_{\text{gain}}=1$. Thus, this analysis did not test whether low and high gain produced the same absolute camera counts; the absolute count rates were expected to differ. Instead, it tested whether the gain change preserved the relative 785-to-850 spectral response. The exposure-response fits indicated that this relative response was approximately gain invariant, while the residual departure of $K_{\text{gain}}$ from unity was retained as an explicit correction rather than assumed to be zero.

The 850 nm channel was retained as the reference channel. Accordingly, the low-gain zero-thickness calibration images were gain harmonized as

$${\overset{˜}{S}}_{785,0,c}(p)=\frac{S_{785,0,c}(p)}{K_{\text{gain}}},\;{\overset{˜}{S}}_{850,0,c}(p)=S_{850,0,c}(p),$$ (25)

where c denotes fluorophore concentration. This operation corrects only the relative wavelength dependence of the gain response. Any multiplicative gain component common to both channels cancels in the subsequent two-channel ratio. If $K_{\text{gain}}=1$, no gain correction is applied.

##### 2.8.1.2 Same-filter zero-thickness channel normalization

Following the channel-normalization strategy of García et al., each spectral image was normalized by a concentration-matched zero-thickness calibration acquired with the same filter. The calibration scalar for each wavelength and concentration was defined from the median of the corresponding gain-harmonized, dark-subtracted calibration ROI:

$$C_{785,c}=\underset{p\in \Omega_{0,c}^{+}}{\text{median}}[{\overset{˜}{S}}_{785,0,c}(p)]=\frac{1}{K_{\text{gain}}}\underset{p\in \Omega_{0,c}^{+}}{\text{median}}[S_{785,0,c}(p)],\;C_{850,c}=\underset{p\in \Omega_{0,c}^{+}}{\text{median}}[{\overset{˜}{S}}_{850,0,c}(p)]=\underset{p\in \Omega_{0,c}^{+}}{\text{median}}[S_{850,0,c}(p)].$$ (26)

For each nonzero-thickness measurement i, the signal in each channel was first divided by its same-filter calibration scalar,

$$N_{785,i}(p)=\frac{S_{785,i}(p)}{C_{785,c_{i}}},\;N_{850,i}(p)=\frac{S_{850,i}(p)}{C_{850,c_{i}}},$$ (27)

where $c_{i}$ is the concentration associated with measurement i. The analytical ratio was then calculated from the two independently normalized channels:

$$R_{\mathrm{cal},i}(p)=\frac{N_{785,i}(p)}{N_{850,i}(p)}=\frac{S_{785,i}(p)/C_{785,c_{i}}}{S_{850,i}(p)/C_{850,c_{i}}}.$$ (28)

Equivalently, in the logarithmic domain,

$$\ln\;R_{\mathrm{cal},i}(p)=\ln(\frac{S_{785,i}(p)}{S_{850,i}(p)})-\ln(\frac{C_{785,c_{i}}}{C_{850,c_{i}}}).$$ (29)

This normalization removes concentration- and channel-dependent multiplicative intensity scaling before the analytical model is applied. No explicit rescaling was applied to account for the difference between the 3 ms zero-thickness calibration exposure and the 10 ms tissue exposure. This treatment assumes that, following matched-dark subtraction and the relative spectral-gain harmonization described above, the detector response is linear and multiplicative with exposure over the relevant unsaturated signal range, with negligible residual additive offsets. Under this model,

$$S_{\lambda ,i}(p)=t_{i}Q_{\lambda ,i}(p),\;C_{\lambda ,c}=t_{0}Q_{\lambda ,0,c},$$ (30)

where $t_{i}=10\;\mathrm{ms}$ is the tissue-image exposure, $t_{0}=3\;\mathrm{ms}$ is the zero-thickness calibration exposure, and Q denotes the corresponding exposure-normalized tissue signal or calibration scalar. Because the 785 and 850 nm channels use a common exposure time within each tissue pair and within each calibration pair, the exposure factors cancel algebraically from the channel-normalized ratio:

$$R_{\mathrm{cal},i}(p)=\frac{t_{i}Q_{785,i}(p)/\left[t_{0}Q_{785,0,c_{i}}\right]}{t_{i}Q_{850,i}(p)/\left[t_{0}Q_{850,0,c_{i}}\right]}\;=\frac{Q_{785,i}(p)/Q_{785,0,c_{i}}}{Q_{850,i}(p)/Q_{850,0,c_{i}}}.$$ (31)

Thus, the omission of an additional exposure-time correction follows from the assumed linear-response model rather than from an assumption that the 3 and 10 ms acquisitions produce equivalent absolute signal levels.

Importantly, the zero-thickness calibration is used only for channel-by-channel intensity normalization. It does not replace the analytical intercept $\ln(D_{850}/D_{751})$. The pixelwise analytical depth estimate was therefore calculated as

d^ana,i(p)=ln Rcal,i(p)−ln(D850/D751)(1/δ850)−(1/δ751)=ln Rcal,i(p)−bphysmana. (32)

#### 2.8.2 Empirical scalar-ratio baseline

The empirical baseline used the same 785 nm notch and 850 nm long-pass channels, 785-over-850 ratio orientation, temporal averaging, matched dark subtraction, 81×78-pixel ROI, and valid-pixel criterion defined above. All empirical tissue measurements were evaluated using a fixed 30 ms exposure in both channels and were dark-subtracted using exposure-matched 30 ms dark frames. The relative-gain harmonization and same-filter zero-thickness normalization described above were specific to the analytical branch and were not applied to the empirical baseline. Instead, the empirical method learned the complete conversion from the measured log-ratio to depth directly from tissue measurements of known thickness.

For each measurement i, the pixelwise log-ratio and corresponding median ROI-level scalar feature were defined as

$$x_{i}(p)=\ln\left(\frac{S_{785,i}(p)}{S_{850,i}(p)}\right),\;x_{i}^{\mathrm{med}}=\underset{p\in \Omega_{i}^{+}}{\text{median}}[x_{i}(p)].$$ (33)

Thus, each training measurement contributed one median ROI log-ratio feature, $x_{i}^{\mathrm{med}}$, obtained from the same valid ROI used for the analytical baseline. Because the 785 and 850 nm images were acquired at the same 30 ms exposure within each measurement, any common multiplicative exposure factor cancels in their ratio. Any fixed multiplicative difference between the two channels under this acquisition condition appears as an additive offset in the logarithmic feature and is therefore absorbed into the empirically fitted intercept.

The empirical model was evaluated using the same held-out test thicknesses used throughout the comparison,

$$D_{\mathrm{eval}}=\{2.2,2.5,4.0,6.0\}\;\mathrm{mm}.$$ (34)

For each held-out thickness $d_{h}\in D_{\mathrm{eval}}$, all measurements acquired at $d_{h}$ were excluded from the regression fit. The empirical training thicknesses for that split were

$$D_{\text{train}}(d_{h})=(D_{\mathrm{eval}}\backslash \{d_{h}\})\cup \{8.5\;\mathrm{mm}\}.$$ (35)

The 8.5 mm condition was therefore retained as an additional known-thickness training point in every empirical regression, but it was not treated as a held-out test thickness and was not included directly in the reported prediction-error summaries. A separate regression was fitted for each ICG concentration c and each held-out thickness $d_{h}$. Let

$$I_{c,d_{h}}^{\text{train}}=\{j:c_{j}=c,d_{j}\in D_{\text{train}}(d_{h})\},$$ (36)

denote the concentration-matched training measurements available in a given split. Tissue depth was modeled directly as an affine function of the median ROI log-ratio,

$$d_{j}=a_{c,d_{h}}x_{j}^{\mathrm{med}}+b_{c,d_{h}}+\epsilon_{j},\;j\in I_{c,d_{h}}^{\text{train}},$$ (37)

where $a_{c,d_{h}}$ is the empirical ratio-to-depth slope, $b_{c,d_{h}}$ is the empirical depth intercept, and $\epsilon_{j}$ is the regression residual. Both parameters were estimated by ordinary least squares using only the non-held-out training measurements:

(a^c,dh,b^c,dh)=arg mina,b∑j∈Ic,dhtrain[dj−(axjmed+b)]2. (38)

For a test measurement i acquired at the held-out thickness $d_{h}$, the same median ROI log-ratio feature $x_{i}^{\mathrm{med}}$ defined in Eq. (33) was computed from the valid held-out ROI. The fitted affine predictor for concentration c and held-out thickness $d_{h}$ was defined as

f^c,dh(x)=a^c,dhx+b^c,dh. (39)

The ROI-level empirical depth prediction used to calculate the reported prediction errors and RMSE values was then

d^emp,i=f^ci,dh(ximed)=a^ci,dhximed+b^ci,dh. (40)

Thus, the same median ROI log-ratio statistic was used during both regression fitting and held-out prediction.

Unlike the analytical baseline, neither the empirical slope nor the empirical intercept was specified using literature absorption, reduced-scattering, diffusion, or penetration depth parameters. Instead, both parameters were re-estimated from the measured known-thickness training data for every concentration and leave-one-thickness-out split. Consequently, no measurement acquired at the held-out thickness contributed to the regression used to predict that thickness, whereas the 8.5 mm condition served only as an additional training anchor.

## 3 Results

### 3.1 Wavelength-Dependent Radial Spread Profiles

**Fig. 2:**
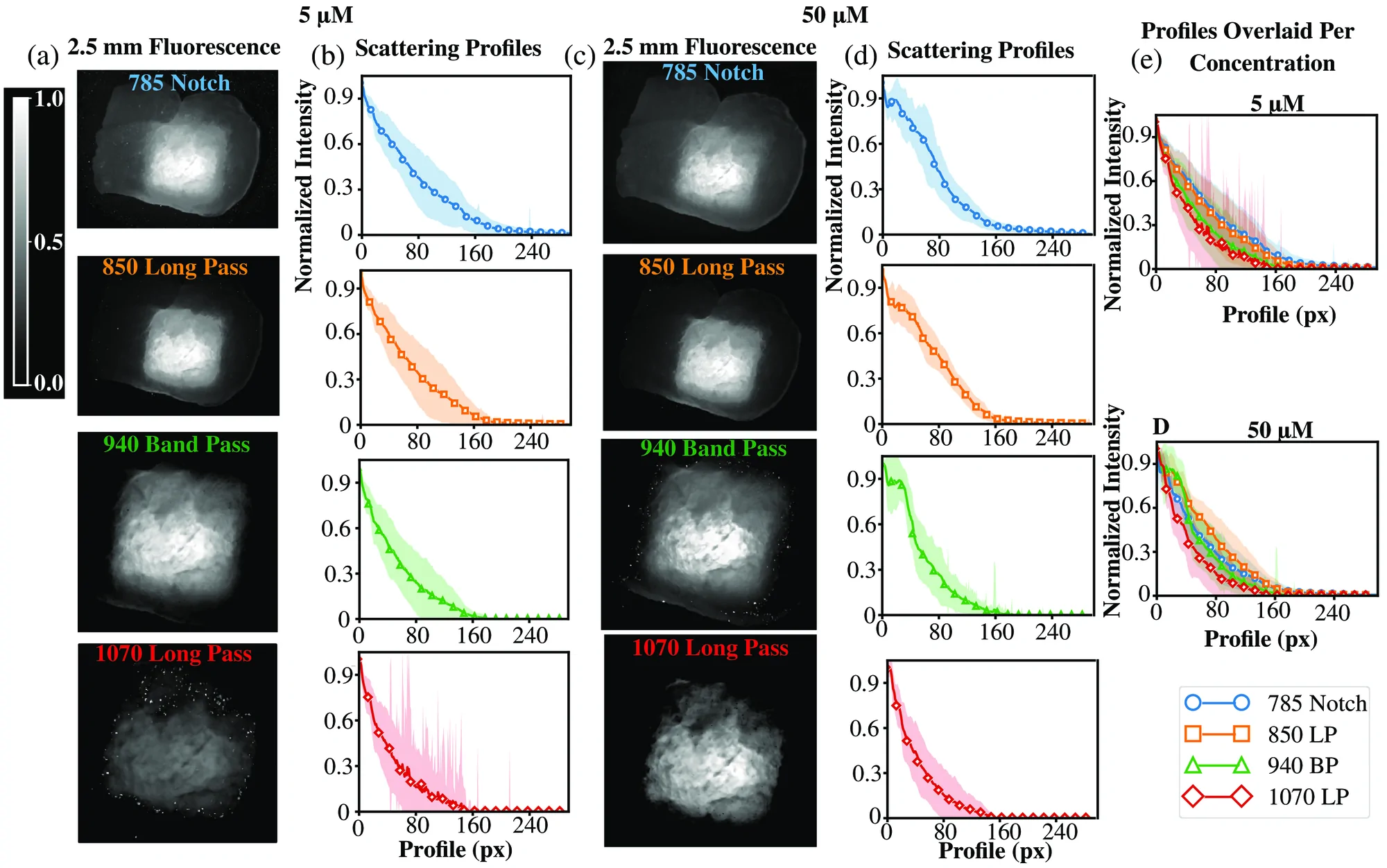
Representative training data for the 2.5 mm chicken-breast condition acquired from 5 and 50  μM ICG phantoms. High-dynamic-range (HDR) reconstructions are shown for four spectral filters: 785 nm notch, 850 nm long-pass, 940 nm band-pass, and 1070 nm long-pass. Radial spread profiles were extracted from concentric annuli centered on the brightest image location, and each profile value corresponds to the mean intensity within one annulus. Shaded regions denote ±1 standard deviation. 5  μM phantom: (a) HDR images and (b) corresponding peak-normalized radial spread profiles. 50  μM phantom: (c) HDR images and (d) corresponding peak-normalized radial spread profiles. (e) Overlaid radial spread profiles for the four spectral bands at 5 and 50  μM.

Figure 2 shows representative multispectral HDR images and peak-normalized radial spread profiles for the 2.5 mm tissue condition. Across all spectral bands, the profiles decay monotonically with radius. Changing ICG concentration from 5 to 50  μM primarily changes signal magnitude, whereas the overall profile shape remains similar within each spectral band. In contrast, the dependence on detection wavelength is pronounced: the 940 nm band-pass and 1070 nm long-pass channels exhibit more compact profiles than the 785 nm notch and 850 nm long-pass channels, approaching baseline by ∼160  pixels, while the shorter-wavelength channels remain broader over a larger radial extent. This trend is also evident in the overlaid profiles in Fig. 2(e).

A second observation from Fig. 2 is that the shorter-wavelength HDR images preserve more visible tissue texture than the longer-wavelength images. Correspondingly, the 785 nm notch and 850 nm long-pass profiles exhibit broader tails than the 940 nm band-pass and 1070 nm long-pass profiles. These results show that the same buried ICG source generates wavelength-dependent spread profiles, providing complementary multispectral information for depth reconstruction.

### 3.2 Depth Dependence across the Training Set

**Fig. 3:**
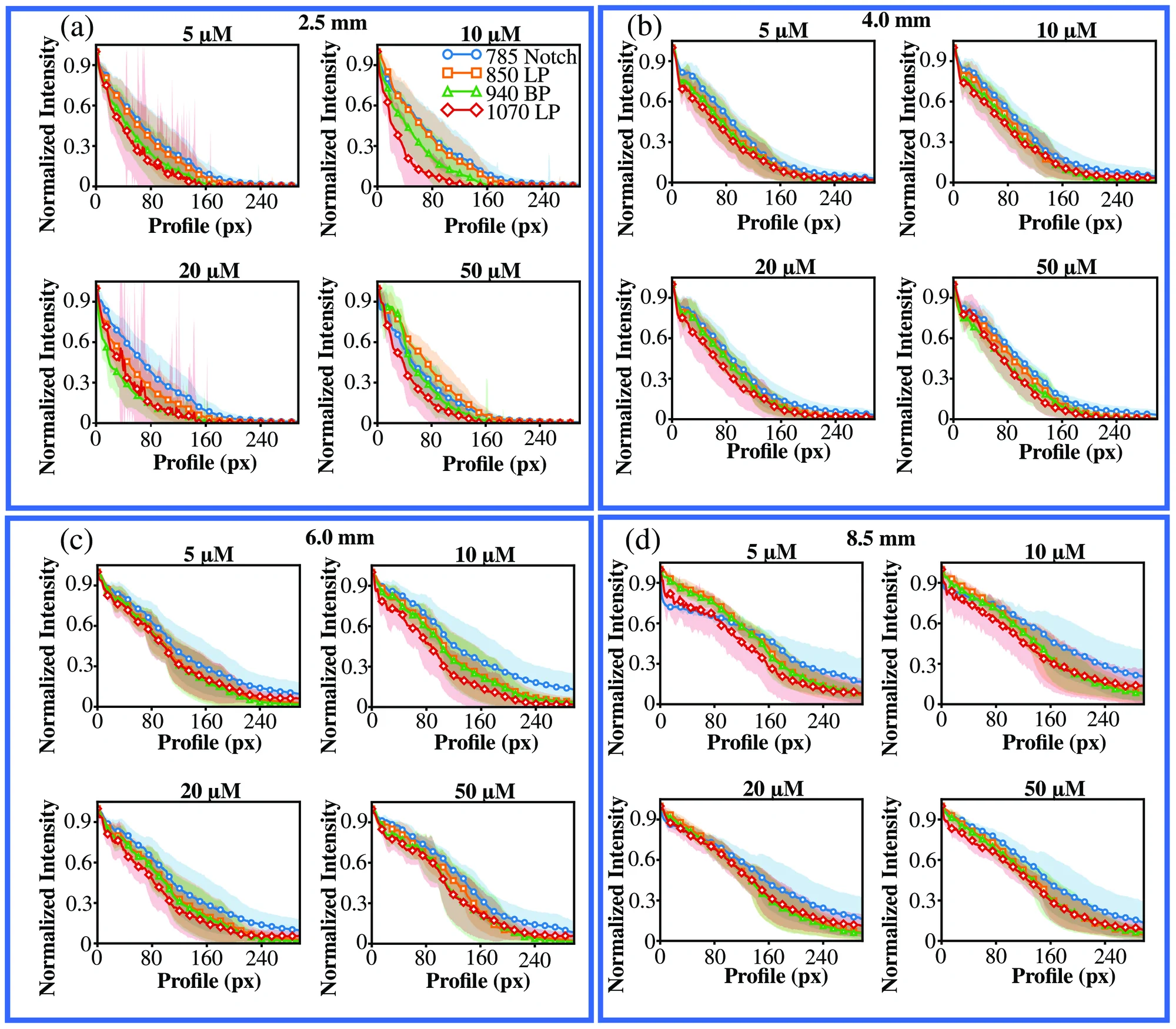
Representative peak-normalized radial spread profiles from the training dataset, shown for four of the five acquired tissue thicknesses: 2.5 mm (a), 4.0 mm (b), 6.0 mm (c), and 8.5 mm (d). For each thickness, panels corresponding to 5, 10, 20, and 50  μM ICG phantoms are shown and include profiles acquired with the 785 nm notch, 850 nm long-pass, 940 nm band-pass, and 1070 nm long-pass filters.

**Fig. 4:**
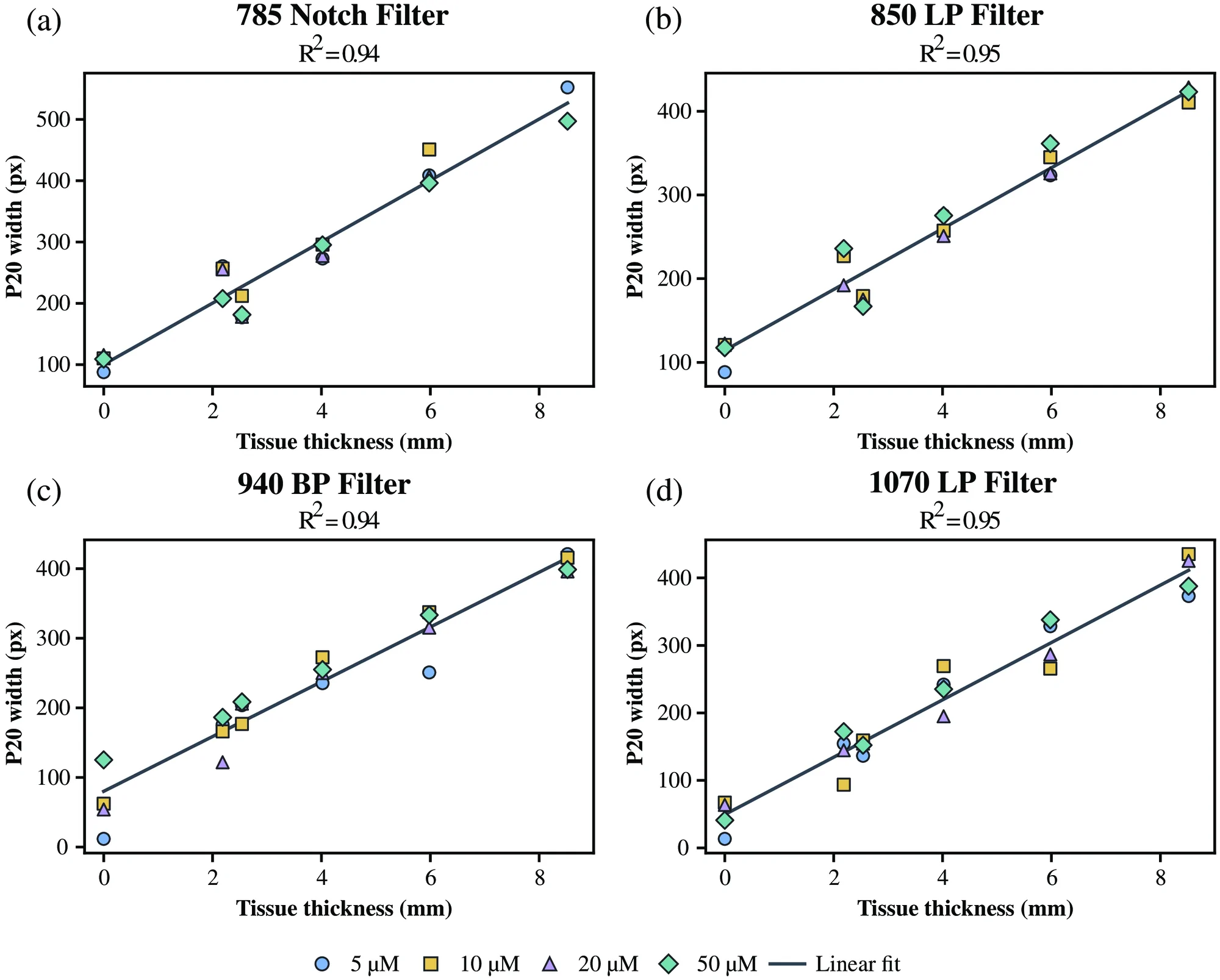
Quantification of radial spread-profile broadening with tissue thickness. P20 width was defined as the full profile width at 20% of the peak-normalized intensity, $w_{20}=2r_{20}$, where $r_{20}$ is the first post-peak radial crossing of the 20% intensity level. P20 width values were pooled across ICG concentrations within each spectral filter, including the zero-thickness (no-tissue) condition, and plotted as a function of tissue thickness for (a) the 785 nm notch filter, (b) the 850 nm long-pass filter, (c) the 940 nm band-pass filter, and (d) the 1070 nm long-pass filter. Solid lines denote ordinary least-squares linear fits. The resulting $R^{2}$ values were greater than or equal to 0.94 across all filters, demonstrating a strong correlation between radial spread-profile width and overlying tissue thickness across spectral bands.

**Fig. 5:**
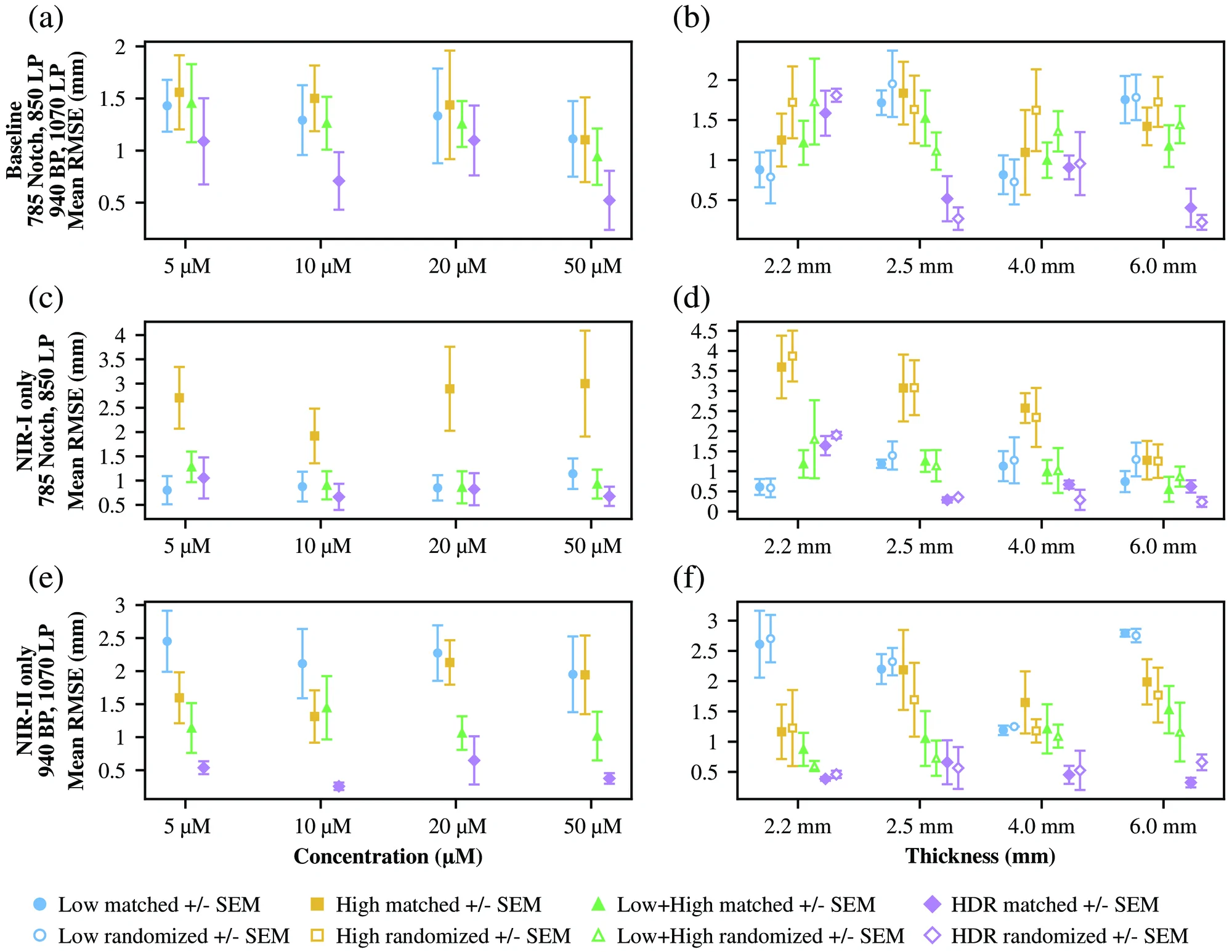
Depth-reconstruction accuracy under four different exposure conditions and three different spectral band ablations. Exposure configurations include low-exposure images only $\left(1<t_{e}\leq 50\;\mathrm{ms}\right)$, high-exposure images only $\left(t_{e}\geq 200\;\mathrm{ms}\right)$, a paired low+high exposure HDR reconstruction, and baseline HDR reconstruction using the complete exposure stack. Each exposure condition was applied to all spectral band ablations. Panels (a) and (b) show all four spectral filters are included, corresponding to the 785 nm notch, 850 nm long-pass, 940 nm band-pass, and 1070 nm long-pass channels. Panels (c) and (d) show shorter-wavelength/NIR-I channels only, corresponding to the 785 nm notch and 850 nm long-pass channels. Panels (e) and (f) show Longer-wavelength/NIR-II channels only, corresponding to the 940 nm band-pass and 1070 nm long-pass channels. Left column: mean RMSE ± SEM reported for each ICG concentration under concentration-matched testing, averaged across the four held-out thicknesses of 2.2, 2.5, 4.0, and 6.0 mm. Right column: mean RMSE ± SEM reported for each held-out tissue thickness averaged across concentration-matched and concentration-randomized conditions.

**Fig. 6:**
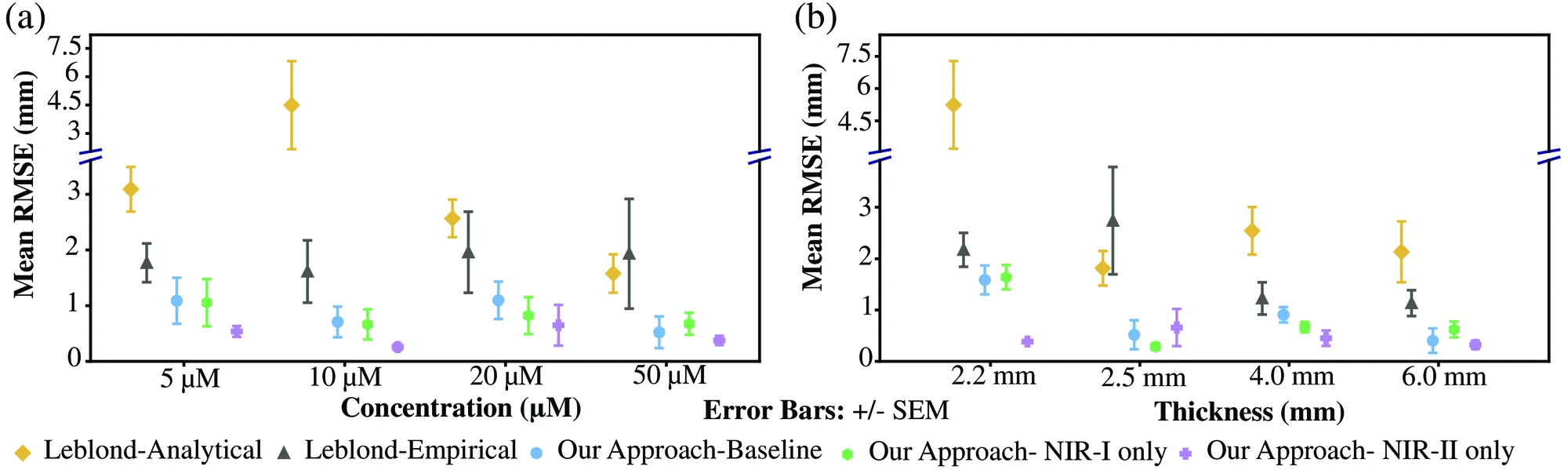
Comparison of depth-reconstruction performance between the proposed multispectral spread-profile dictionary method and analytical and empirically calibrated implementations of a scalar dual-emission fluorescence-ratio method. The analytical scalar-ratio method applies the dual-emission model of Leblond et al., which relates the logarithm of the fluorescence intensity ratio at two emission wavelengths to source depth under a diffusion-theory description of photon transport in homogeneous turbid media, as presented in Eq. (1) of García et al.^5^^,^^12^ The empirical scalar-ratio baseline uses the same ROI-level median log-ratio statistic during both training and held-out testing: the slope and intercept are estimated by linear regression from the median log-ratios of the training ROIs within each leave-one-thickness-out regime and are then applied to the median log-ratio of the held-out ROI. The proposed spread-profile method is shown using all four spectral channels (all-filter baseline), the shorter-wavelength/NIR-I subset comprising the 785 nm notch and 850 nm long-pass channels, and the longer-wavelength/NIR-II subset comprising the 940 nm band-pass and 1070 nm long-pass channels. (a) Mean depth RMSE (±) SEM for each ICG concentration under concentration-matched testing, averaged across the held-out tissue thicknesses of 2.2, 2.5, 4.0, and 6.0 mm. (b) Mean depth RMSE (±) SEM for each held-out tissue thickness, averaged across the evaluated ICG concentrations under concentration-matched testing. Broken y-axes are used to display the larger errors of the analytical scalar-ratio baseline.

Figure 3 summarizes peak-normalized radial spread profiles for 5, 10, 20, and 50  μM ICG phantoms acquired with the 785 nm notch, 850 nm long-pass, 940 nm band-pass, and 1070 nm long-pass filters at tissue thicknesses of 2.5, 4.0, 6.0, and 8.5 mm. The 2.2 mm condition is omitted from Fig. 3 for display layout only; it is included in the P20 width regression of Fig. 4 and in all reconstruction analyses reported in Figs. 5 and 6. Across concentrations and spectral bands, the profile width increased systematically with tissue thickness: shallower conditions yielded more compact profiles, whereas thicker tissue produced broader fluorescence spread. We further quantified this trend by fitting P20 spread-profile width as a function of tissue thickness using ordinary least-squares linear regression for each spectral band in Fig. 4. The resulting fits showed strong correlations across both NIR-I and NIR-II channels, with $R^{2}$ values greater than or equal to 0.94 for all filters, supporting that radial profile broadening is associated with increasing overlying tissue thickness.

The wavelength dependence observed in Fig. 2 is preserved across the training set. At shallower thicknesses, particularly 2.5 and 4.0 mm, the 1070 nm long-pass profiles remain the most compact, whereas the 785 nm notch and 850 nm long-pass profiles remain broader over a longer radial distance. This separation persists at 6.0 mm but becomes less distinct at 8.5 mm, where the variability increases, especially in the 1070 nm channel. Despite this reduction in spectral separation at the largest thickness, the profiles remain depth-dependent across filters and concentrations, supporting their use as dictionary elements for the inverse model. The training thicknesses used to construct the final prediction errors include the 8.5 mm training case but omit this thickness as a held-out test case due to lowered SNR across spectral bands at this thickness.

### 3.3 Depth-Reconstruction Performance

Prediction errors across concentration, tissue thickness, exposure condition, and spectral subset are summarized in Fig. 5. Using all four filters, full HDR reconstruction produced the lowest concentration-matched RMSE among the four exposure configurations when errors were averaged across the four held-out thicknesses for each ICG concentration. The all-filter HDR RMSE values were 1.09±0.41, 0.71±0.28, 1.10±0.34, and 0.52±0.28  mm at 5, 10, 20, and 50  μM, respectively. These were lower than the corresponding low-exposure-only values of 1.43±0.25, 1.29±0.34, 1.33±0.45, and 1.11±0.36  mm; high-exposure-only values of 1.56±0.36, 1.50±0.32, 1.44±0.52, and 1.10±0.41  mm; and paired low+high exposure values of 1.45±0.37, 1.26±0.25, 1.25±0.22, and 0.94±0.27  mm. These results support the use of HDR reconstruction for extracting spread profiles when performance is summarized across concentrations.

When errors were summarized by held-out thickness, full HDR reconstruction with all four filters was most beneficial at 2.5 and 6.0 mm. At 2.5 mm, the all-filter HDR RMSE was 0.52±0.28  mm under concentration-matched testing and 0.27±0.14  mm under concentration-randomized testing. At 6.0 mm, the corresponding values were 0.40±0.24 and 0.22±0.09  mm. At 4.0 mm, HDR remained lower than the high-exposure-only and paired low + high conditions but did not outperform the low-exposure-only condition, with HDR errors of 0.91±0.15  mm matched and 0.96±0.39  mm randomized compared with low-exposure-only errors of 0.82±0.24 and 0.73±0.28  mm. The main exception to the HDR trend occurred at the 2.2 mm held-out thickness, where all-filter HDR produced elevated errors of 1.59±0.28  mm matched and 1.81±0.08  mm randomized. For this same 2.2 mm condition, low-exposure-only reconstruction remained sub-millimeter, with errors of 0.88±0.22  mm matched and 0.79±0.33  mm randomized, whereas the high-exposure-only and paired low+high conditions yielded 1.25±0.33/1.72±0.45  mm and 1.22±0.28/1.73±0.54  mm for matched/randomized testing, respectively.

The spectral-band ablation suggests that the increased all-filter HDR error at 2.2 mm was driven primarily by the shorter-wavelength channels. With NIR-I-only HDR reconstruction, the 2.2 mm errors were 1.64±0.24  mm matched and 1.90±0.08  mm randomized. In contrast, using low-exposure NIR-I-only images reduced the 2.2 mm errors to 0.61±0.20  mm matched and 0.59±0.23  mm randomized.

The most consistent HDR performance was obtained with the longer-wavelength 940 and 1070 nm channels. When RMSE was averaged across the four held-out thicknesses, NIR-II HDR reconstruction produced concentration-matched values of 0.54±0.10, 0.26±0.06, 0.65±0.36, and 0.38±0.08  mm at 5, 10, 20, and 50  μM, respectively. When summarized by held-out thickness, NIR-II HDR reconstruction remained sub-millimeter at every tested thickness: 0.39±0.03, 0.66±0.36, 0.45±0.15, and 0.32±0.08  mm for matched testing at 2.2, 2.5, 4.0, and 6.0 mm and 0.46±0.06, 0.56±0.35, 0.53±0.33, and 0.66±0.13  mm for randomized testing. Thus, although the dictionary is sensitive to exposure selection, full HDR reconstruction using the longer-wavelength channels provided the most robust sub-millimeter depth estimation across the expanded held-out thickness analysis.

To place these results in the context of established scalar intensity-ratio methods, we next compared the proposed spread-profile dictionary approach with analytical and empirically calibrated implementations of the scalar dual-emission fluorescence-ratio method of Leblond et al.^5^^,^^12^ (Fig. 6). For this direct comparison, all methods were summarized under the same concentration-matched leave-one-thickness-out evaluation framework over the 2.2, 2.5, 4.0, and 6.0 mm held-out conditions. When RMSE was averaged across the four held-out thicknesses for each ICG concentration [Fig. 6(a)], the analytical and empirical scalar-ratio baselines yielded ranges of 1.58 to 4.49 mm and 1.61 to 1.96 mm, respectively. In comparison, the full-HDR spread-profile method yielded RMSE ranges of 0.52 to 1.10 mm using all four filters, 0.66 to 1.05 mm using the NIR-I-only subset, and 0.26 to 0.65 mm using the NIR-II-only subset. When RMSE was instead averaged across the four ICG concentrations for each held-out thickness [Fig. 6(b)], the analytical and empirical scalar-ratio baselines ranged from 1.81 to 5.24 mm and 1.13 to 2.74 mm, respectively, whereas the corresponding spread-profile ranges were 0.40 to 1.59 mm, 0.29 to 1.64 mm, and 0.32 to 0.66 mm for the all-filter, NIR-I-only, and NIR-II-only HDR configurations. The empirical scalar-ratio results were lower than the analytical benchmark for most, but not all, concentration- and thickness-averaged conditions, indicating that the relative performance of the two scalar-ratio implementations depended on the evaluated condition. Across all displayed concentration- and thickness-averaged conditions, however, the spread-profile configurations yielded lower RMSE than the empirical scalar-ratio model, with the most consistently low errors obtained using the NIR-II-only configuration, which remained below 0.7 mm in both comparisons.

### 3.4 Preliminary Clinical Comparison with Sentinel Lymph Nodes

**Fig. 7:**
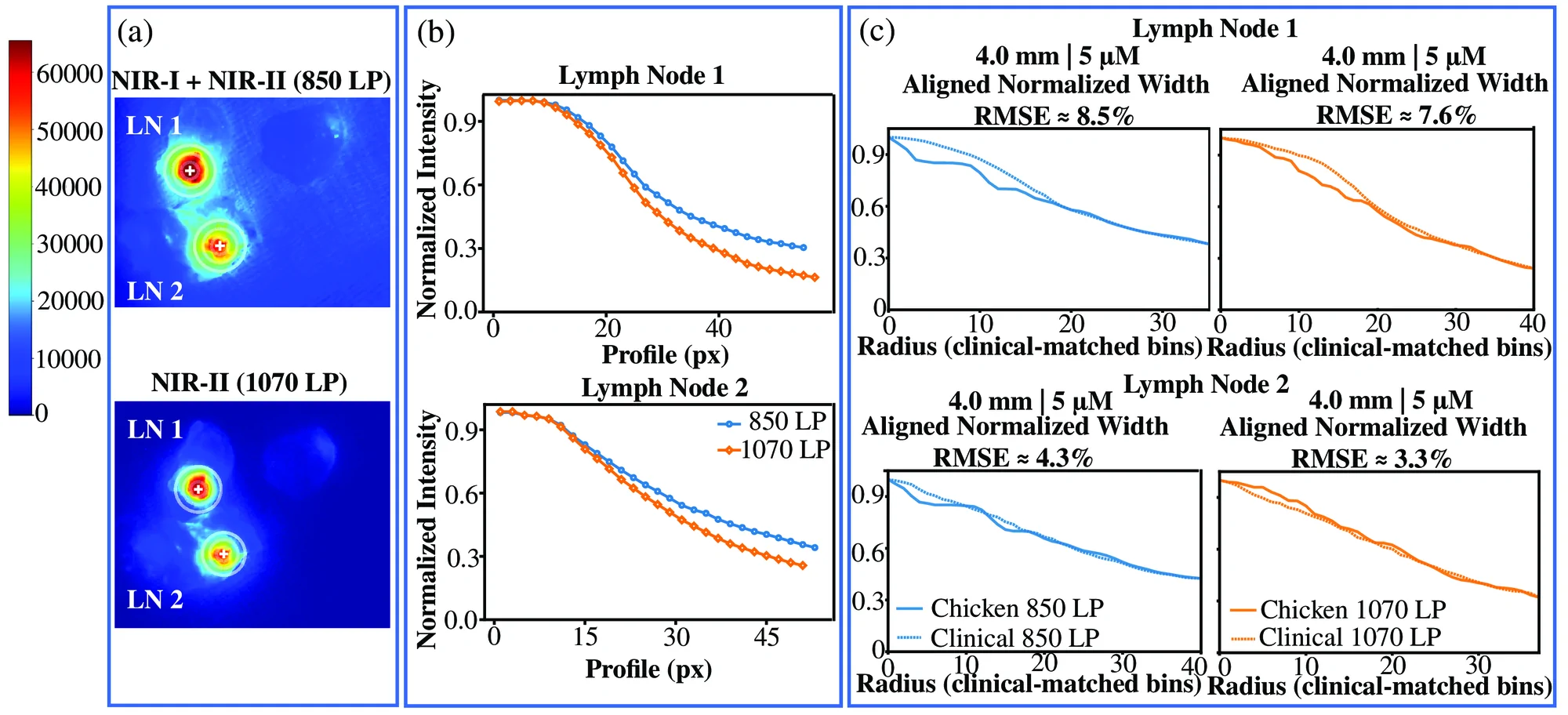
Preliminary *ex vivo* clinical comparison using two ICG-localized sentinel lymph nodes (SLNs) from a breast cancer specimen. (a) Clinical images acquired with the 850 nm long-pass and 1070 nm long-pass filters. (b) Peak-normalized radial spread profiles from the 850 nm long-pass and 1070 nm long-pass channels for SLN 1 and SLN 2; white annuli indicate representative ROIs used for profile extraction. (c) Closest-matching chicken-breast phantom and clinical SLN profiles for the 850 nm long-pass and 1070 nm long-pass channels, together with normalized-width RMSE values. For spread-profile comparison, chicken-breast profiles were truncated to the observable clinical intensity and then resampled to the clinical profile length. The comparison was performed over the normalized intensity range corresponding to the decay region of the profile, excluding values above 80% peak intensity, while the plotted overlays display the full peak-trimmed and normalized spread functions.

The clinical examples in Fig. 7 were obtained in an *ex vivo* lymphatic mapping workflow from a patient with breast cancer undergoing mastectomy with axillary node evaluation. For lymphatic mapping, a 25 mg vial of ICG was reconstituted in 25 mL of sterile water (1  mg/mL), and 10 mg of this solution was administered at four periareolar injection sites, followed by 3 to 5 min of gentle massage to promote lymphatic uptake. Patients also received radiocolloid tracer per institutional standard of care for gamma-probe mapping. The resected breast specimen and associated lymph nodes were imaged immediately after removal. NIR fluorescence was first used to localize the nodes, after which the surrounding tissue was carefully dissected to expose each node for multispectral imaging. Histopathology with hematoxylin and eosin (H&E) staining served as the clinical reference standard.

Figure 7 shows two exposed sentinel lymph nodes imaged with the 850 nm long-pass and 1070 nm long-pass filters. In both nodes, the 1070 nm images exhibit lower detected intensity and a more compact apparent spread than the corresponding 850 nm images. This behavior is reinforced by the radial spread profiles in Fig. 7(b), where the 1070 nm curves decay to baseline over a shorter radial distance than the 850 nm curves for both SLN 1 and SLN 2. Thus, both clinical nodes preserve the same qualitative wavelength-dependent narrowing observed in the phantom data.

A comparison between the two nodes also shows that their profile widths are broadly similar within a given spectral band. SLN 1 appears slightly more compact than SLN 2, but both nodes follow the same spectral ordering, with the 1070 nm profile narrower than the 850 nm profile. Pathologic review of the corresponding H&E sections showed similar distances from each SLN to the tissue border, ∼2.0±0.2  mm for both nodes. Although histologic border distances do not map directly onto intact tissue geometry because of sectioning and tissue processing, the agreement between the two nodes is reassuring because their optical spread profiles are also broadly similar.

To assess whether the phantom-derived profiles captured clinically relevant profile shapes, each clinical SLN profile was compared with the closest-matching chicken-breast phantom profile. As currently labeled in Fig. 7(c), the best-match normalized-width RMSE values were 7.6% and 3.3% for SLN 1 and SLN 2, respectively, in the 1070 nm channel and 8.5% and 4.3% in the 850 nm channel. These comparisons indicate that the phantom dictionary reproduces the major features of the clinical spread profiles, while also showing that the degree of phantom-to-clinical agreement differs somewhat between nodes and spectral bands.

Because independent ground-truth optical depth was not available for these specimens, Fig. 7 should be interpreted as preliminary translational support rather than as a direct validation of clinical depth estimation. Nevertheless, the preservation of wavelength-dependent profile narrowing in both SLNs supports the translational relevance of the spread-profile framework in freshly resected clinical tissues.

## 4 Discussion and Conclusion

The results of this study support the central premise that fluorescence depth information can be encoded not only in scalar intensity ratios but also in the wavelength dependence of the full spatial spread profile. Across the phantom experiments, longer-wavelength channels produced more compact radial spread profiles than shorter-wavelength channels, and these profile differences varied systematically with tissue thickness. This observation was further supported by the P20 width analysis in Fig. 4, which showed strong linear relationships between peak-normalized radial spread-profile width and tissue thickness across spectral filters, with $R^{2}$ values greater than or equal to 0.94. Using the full depth-encoding radial profiles as dictionary elements, the inverse model recovered tissue depth with sub-millimeter RMSE under the concentration-matched and concentration-randomized conditions. In this sense, the present approach extends earlier dual-wavelength depth-estimation methods by using the full radial profile as the depth-bearing observable rather than collapsing each measurement to a single ratio value.^4^^–^^7^

To determine whether retaining spatial information improved depth reconstruction relative to established scalar observables, we compared the spread-profile dictionary method with analytical and empirically calibrated implementations of the dual-emission fluorescence-ratio approach in Fig. 6. The empirical scalar-ratio model provides the more informative dataset-specific comparator because its slope and intercept were learned from measured training-tissue data and may therefore absorb fixed passband and system-response effects. Across these comparisons, the spread-profile configurations generally produced lower RMSE than the empirical scalar-ratio model, with the strongest performance obtained using the longer-wavelength channels. Whether this numerical improvement represents a clinically meaningful advantage will depend on the procedure-specific depth precision required. Profile-based estimation may be most beneficial when millimeter-scale differences affect tissue preservation or margin decisions, whereas an empirically calibrated scalar-ratio method may be sufficient for applications requiring only coarser localization. These potential uses require prospective validation under clinically representative conditions.

The analytical result should be interpreted as a literature-parameterized benchmark rather than as a definitive head-to-head comparison of the Leblond model. The 785 nm notch and 850 nm long-pass measurements integrate fluorescence over broad spectral response ranges, whereas the present implementation used published point optical-property values at 751 and 850 nm as wavelength proxies.^19^^,^^20^ A rigorous broadband formulation would instead integrate the ICG emission spectrum, filter transmission, wavelength-dependent tissue optical properties, and detector sensitivity over the complete response of each channel. Prior sensitivity analyses showing substantial depth bias under optical-property mismatch further emphasize this limitation.^12^ Although the wavelength-dependent gain mismatch was corrected before zero-thickness normalization, residual analytical error may still reflect uncertainty in the broadband optical-property approximation, forward-model assumptions, and gain harmonization, in addition to limitations of the scalar-ratio observable itself.

An important distinction between the proposed method and the analytical ratio model is that the proposed method is calibration-driven rather than fully analytical. By learning the depth relationship directly from measured profiles, the dictionary avoids requiring a fully specified photon-transport model and may absorb fixed instrument and passband effects. This calibration may contribute to the observed robustness under concentration randomization, but it also means that the learned profile-to-depth relationship may not transfer directly across tissues, imaging systems, or acquisition geometries, and changes in these conditions may require recalibration. Two observations bound this dependence in the present dataset: depth reconstruction remained sub-millimeter when the dictionary and the test profiles were drawn from different ICG concentrations, indicating that the recovered relationship is not specific to fluorophore concentration, and the clinical sentinel lymph nodes in Fig. 7 reproduced the same wavelength-dependent profile narrowing in freshly resected human tissue rather than in the chicken-breast calibration medium.

The observed spectral ordering is also physically consistent with the expected wavelength dependence of light transport in tissue. Because reduced scattering generally decreases with increasing wavelength, longer-wavelength fluorescence should experience less diffuse broadening than shorter-wavelength fluorescence, leading to a more compact apparent profile at the tissue surface. The profile narrowing observed in the 940 and 1070 nm channels relative to the 785 and 850 nm channels is consistent with this expectation. At the same time, the separation between NIR-I and NIR-II profiles decreased at the largest tested thickness, particularly in the 1070 nm channel, suggesting that the long-wavelength advantage is not unlimited. In practice, the observed spread profile reflects a combination of tissue scattering, absorption, filter bandwidth, detector sensitivity, and background contributions. Thus, although longer wavelengths generally produced more compact profiles in our data, the degree of separation between spectral bands will remain system-dependent.^14^^,^^16^^,^^17^

Within this calibration framework, exposure selection remains important because area normalization does not fully remove the effects of SNR, background, and saturation on the recovered profile shape. Full HDR reconstruction generally improved the concentration-averaged all-filter results because it combines information across low- and high-exposure images and favors unsaturated high-SNR measurements. However, the 2.2 mm condition revealed an important shallow-depth failure mode: when the shorter-wavelength channels were included, all-filter HDR errors increased, whereas low-exposure NIR-I reconstruction and HDR reconstruction using only the longer-wavelength channels remained sub-millimeter. The HDR algorithm explicitly suppresses detector values below the noise threshold and above the saturation threshold and weights mid-range, higher-SNR exposures (Appendix A), but near-saturated pixels below the saturation threshold may still influence the recovered profile shape. These results suggest that full HDR reconstruction is beneficial overall, but that shallow-source depth estimation may be most reliable when HDR reconstruction is combined with longer-wavelength channels or with more conservative saturation-margin rejection in the shorter-wavelength channels. Because spread profiles are expressed in detector pixels, the dictionary is also specific to the imaging geometry used for calibration: a change in working distance or lens magnification rescales the measured profile width independently of depth. Expressing profiles in physical units at the sample plane using the known system magnification would remove this dependence and be a prerequisite for transferring a calibrated dictionary between systems.

A related consequence of this calibration dependence is that tissue heterogeneity may weaken the uniqueness of the spread-profile-to-depth relationship. Under the comparatively uniform calibration conditions used here, profiles acquired at a given tissue thickness were assumed to be sufficiently repeatable so that systematic differences between dictionary entries were primarily associated with the amount of overlying tissue. In biological tissue, however, absorption and reduced scattering can vary across tissue types, subjects, and anatomical locations, and these optical properties directly influence the radial distribution of diffusely propagated light.^14^^,^^19^ Consequently, specimens with the same true depth but different optical properties may produce different multispectral spread profiles, whereas different combinations of depth and optical properties may produce similar profiles. Either condition would create a mismatch between the measured profile and the calibration dictionary, potentially increasing systematic depth bias and estimate variability and thereby reducing both accuracy and robustness. Related fluorescence diffuse-optical work has similarly identified heterogeneous optical properties as a source of depth-estimation error. Mitigating this dependence would require local estimation of tissue optical properties at the imaging site, for example, by paired diffuse-reflectance or spatial-frequency-domain measurements, so that dictionary selection can be conditioned on the measured absorption and reduced scattering rather than assumed constant.^21^

The preliminary sentinel lymph node data in Fig. 7 should therefore be interpreted cautiously. These clinical measurements provide translational support for the central wavelength-dependent spread-profile behavior, but they do not yet constitute direct validation of clinical depth estimation because no independent ground-truth node depth was available. In addition, the chicken-breast training data are comparatively homogeneous relative to clinical lymphatic tissue. The degree of phantom-to-clinical agreement was band dependent, with lower normalized-width RMSE values observed in the 1070 nm channel than in the 850 nm channel. Nevertheless, both channels preserved the same qualitative wavelength-dependent narrowing, supporting the translational relevance of the spread-profile framework while indicating that phantom-to-clinical correspondence remains sensitive to spectral band and tissue context. A more rigorous translational study would require specimens or phantoms with independently verified depth, a broader range of tissue compositions, and ideally paired reflectance measurements to separate fluorescence-specific broadening from background tissue contributions.

Clinical translation will also require substantial improvements in computational speed, acquisition hardware, and robustness to intraoperative conditions. The current implementation does not yet provide real-time feedback: HDR reconstruction across all spectral bands required ∼10 to 20 min per held-out test condition, which would represent a substantial interruption to surgical workflow. Restricting the reconstruction to low-exposure NIR-I spread profiles reduced the computation time to ∼1 to 1.5 min while retaining favorable depth accuracy, including sub-millimeter performance at the shallow 2.2 mm condition. This result suggests that selective use of the most informative exposures and spectral channels, together with optimized or GPU-accelerated inference, may provide a more practical route toward intraoperative feedback than full HDR processing.

A clinical NIR-I implementation would nevertheless require strong rejection of excitation light, preferably using filters with high optical density at the excitation wavelength to minimize laser leakage and specularly reflected excitation light. Robustness to ambient operating-room illumination and tissue-surface reflections would additionally require appropriate emission-band selection and background correction. The form of the depth output also requires further consideration. The present method produces one scalar depth estimate for each analyzed fluorescent spread profile rather than a spatially resolved per-pixel depth map, as can be generated by scalar-ratio formulations. Generating a spatially resolved depth map within the present framework would require training and inference at the pixel or radial-bin level against spatially resolved ground-truth depth, which the current phantom geometry does not provide. Whether source-level depth information is sufficient or whether pixelwise depth mapping provides a clinically meaningful advantage will depend on the surgical application and should be evaluated prospectively. Finally, tissue motion and deformation may artificially broaden, compress, or displace the measured spread profile and thereby bias the reconstructed depth. Measurements with the current implementation would therefore need to be obtained during brief periods of relative tissue stability, whereas future systems should incorporate faster snapshot multispectral acquisition, motion detection or registration, and validation under realistic tissue manipulation. Two further conditions distinguish the calibration geometry from the surgical field: the phantom measurements used a planar tissue interface maintained by a glass slide, whereas curved or irregular tissue surfaces vary the source-to-surface distance across the field and would distort the measured radial profile, and the current implementation requires an operator-designated region of interest around a compact fluorescent source rather than operating automatically on the full field.

Frame-based and snapshot multispectral fluorescence cameras are beginning to provide a practical hardware route for implementing methods such as the one presented here. Our approach requires only a limited number of intrinsically co-registered spectral bands under a single excitation wavelength, making it well suited to compact single-chip architectures that can replace sequential filter-wheel acquisition and better support real-time use. Although most current research systems are concentrated in the visible to NIR-I range, they establish the key design principles needed for translation. Expanding these architectures to include NIR-II spectral channels could further strengthen depth-sensitive fluorescence imaging by combining compact hardware, intrinsic spatial co-registration, and longer-wavelength contrast within a clinically practical platform.^22^^–^^25^

At the same time, on-sensor spectral mosaics introduce their own trade-offs. Because each spectral band is sampled on only a subset of pixels, spatial resolution is reduced relative to monochrome imaging, and demosaicing or interpolation becomes necessary. This issue has already been recognized in recent work on hexachromatic surgical sensors, where deep-learning-based demosaicing improved both color and NIR image quality relative to conventional interpolation. For the present method, this trade-off is especially relevant because radial spread-profile extraction depends directly on spatial fidelity near the fluorescence centroid and in the profile tails. Future implementations of the method on pixelated spectral-filter sensors will therefore need sensor-specific calibration, channel-aware preprocessing, and careful evaluation of how demosaicing alters profile width and tail behavior.^26^

Two additional limitations concern depth sampling and source geometry. First, the limited number of calibrated tissue thicknesses means that intermediate depth estimates are inferred from the recovered weight distribution rather than from dense physical sampling of the depth axis, and the present results do not establish performance outside the calibrated range. Second, the spread profile is computed as a ring mean over annuli centered on the refined target centroid and therefore presumes approximate radial symmetry of the apparent fluorescence distribution. The phantom targets used here were compact and approximately symmetric, so this assumption is well matched to the calibration data. For an elongated or highly irregular source, however, the lateral extent of the emitting region contributes to the ring-mean width independently of depth: azimuthal averaging mixes the narrow and broad axes of the source, broadening the measured profile in a manner the dictionary would attribute to additional overlying tissue and thereby biasing the depth estimate deeper. Source shape and depth are therefore not separable in the present one-dimensional formulation. Extending the observable to an orientation-resolved or two-dimensional spread function, or explicitly conditioning the dictionary on source size and eccentricity, would allow these contributions to be disentangled. These limitations should be addressed through denser depth sampling and validation across a broader range of source shapes and sizes.

Overall, the present results suggest that multispectral spread-profile analysis provides a useful intermediate approach between conventional planar fluorescence imaging and more complex tomographic or time-resolved systems. It preserves the simplicity of wide-field acquisition while extracting depth-sensitive information from wavelength-dependent image formation. With further validation and sensor-specific calibration, this strategy may be especially attractive for next-generation surgical imagers that combine compact hardware, on-chip spectral separation, and real-time computational inference.

## 5 Appendix A. HDR Fusion and Centroid Refinement

Let $I_{v,e}^{\text{raw}}(x,y)$ denote the raw image acquired in spectral band v at exposure e, let $D_{v,e}(x,y)$ denote the corresponding dark frame, and let $t_{e}$ denote the exposure time. After dark subtraction and clipping of negative values, the exposure-normalized image was written as

$$r_{v,e}(x,y)=\frac{\max(I_{v,e}^{\text{raw}}(x,y)-D_{v,e}(x,y),0)}{t_{e}}.$$ (41)

The HDR image for spectral band v was then computed as

$$I_{v}(x,y)=\frac{\overset{E}{\underset{e=1}{\sum }}w_{v,e}(x,y)r_{v,e}(x,y)}{\overset{E}{\underset{e=1}{\sum }}w_{v,e}(x,y)},$$ (42)

where $w_{v,e}(x,y)$ is a saturation-aware weight that suppresses clipped pixels and favors unsaturated measurements with a higher signal-to-noise ratio. The per-pixel weight can be written as three distinct parts

w^v,e(x,y)=1−|2zv,e(x,y)−1|, (43)

$$m_{v,e}(x,y)=1_{\left\{J_{v,e}(x,y)>\tau_{\text{low}}\right\}}1_{\left\{J_{v,e}(x,y)<T_{\mathrm{sat}}\right\}},$$ (44)

$$w_{v,e}^{\mathrm{snr}}=\sqrt{t_{e}}.$$ (45)

where $J_{v,e}(x,y)$ denotes the dark-subtracted and temporally averaged detector-domain image and $Z_{v,e}(x,)$ denotes the normalized version of $J_{v,e}(x,y)$ between [0,1]. Next, ${\hat{w}}_{v,e}(x,y)$ places emphasis on mid-range exposures, $m_{v,e}(x,y)$ removes exposures that are too close to the noise floor or are too saturated, and $w_{v,e}^{\mathrm{snr}}$ adds a preference to longer exposures over shorter ones as the SNR is expected to be higher at longer exposures. Finally, the total weight per pixel, $w_{v,e}(x,y)$, can be written as

wv,e(x,y)=w^v,e(x,y)mv,e(x,y)wv,esnr. (46)

After ROI selection, the target center was refined from the local intensity distribution. If ${\overset{˜}{I}}_{v}(x,y)$ denotes the smoothed ROI patch, the centroid estimate can be written as

$$c=\frac{\underset{(x,y)\in \Omega }{\sum }(x,y){\overset{˜}{I}}_{v}(x,y)}{\underset{(x,y)\in \Omega }{\sum }{\overset{˜}{I}}_{v}(x,y)},$$ (47)

where Ω is a small window (700×700) around the local maximum.

## 6 Appendix B. ADMM Formulation

To solve Eq. (4), auxiliary variables z=∇a and s=a were introduced, where s was constrained to the probability simplex

$$\Delta =\{s\in R^{N}:s\geq 0,1^{T}s=1\}.$$ (48)

Using scaled dual variables u and v, the augmented Lagrangian was

$$L(a,z,s,u,v)=\frac{1}{2}\|W(y-Xa){\|}_{2}^{2}+\lambda_{\mathrm{TV}}\|z{\|}_{1}+I_{\Delta }(s)\;+\frac{\rho_{z}}{2}\|\nabla a-z+u{\|}_{2}^{2}+\frac{\rho_{s}}{2}\|a-s+v{\|}_{2}^{2},$$ (49)

where $I_{\Delta }(s)=0$ for s∈Δ and +∞ otherwise.

The ADMM updates were

$$a^{k+1}=\arg\;\underset{a}{\min}\frac{1}{2}\|W(y-Xa){\|}_{2}^{2}+\frac{\rho_{z}}{2}\|\nabla a-z^{k}+u^{k}{\|}_{2}^{2}+\frac{\rho_{s}}{2}\|a-s^{k}+v^{k}{\|}_{2}^{2},$$ (50)

$$z^{k+1}=S_{\lambda_{\mathrm{TV}}/\rho_{z}}(\nabla a^{k+1}+u^{k}),$$ (51)

$$s^{k+1}=\Pi_{\Delta }(a^{k+1}+v^{k}),$$ (52)

$$u^{k+1}=u^{k}+\nabla a^{k+1}-z^{k+1},$$ (53)

$$v^{k+1}=v^{k}+a^{k+1}-s^{k+1},$$ (54)

where $S_{\tau }(q)=\text{sign}(q)\max(|q|-\tau ,0)$ is the elementwise soft-thresholding operator and $\Pi_{\Delta }(\cdot )$ denotes Euclidean projection onto the simplex.

The a-subproblem in Eq. (50) reduces to the linear system

$$(X^{T}W^{T}WX+\rho_{z}\nabla^{T}\nabla +\rho_{s}I)a=X^{T}W^{T}Wy+\rho_{z}\nabla^{T}(z-u)+\rho_{s}(s-v),$$ (55)

which was solved by conjugate gradients.

In the current reconstruction code, W is composed of fixed per-band weights derived from tail noise and profile-shape similarity together with an adaptive robust weight updated from the per-band residuals. The code also supports a per-band amplitude scaling step and a local per-band dilation step to compensate for residual mismatch between training and test spread functions. Therefore, there are eight parameters that adaptively change with each iteration of ADMM in the following order

$$w_{\mathrm{rob},v}\to a\to z\to s\to u\to v\to \gamma_{v}\to \varphi_{v}.$$ (56)

In this sequence $w_{\mathrm{rob}}$ is a robust weight that reduces the influence of spectral spread profiles that create large residuals between test and training datasets, γ denotes the local dilation term that adjusts the radial width of training spread profiles to match test data more accurately, and ϕ reduces overall amplitude differences between training and test spread functions. Let $B_{v}$, $J_{v}$, and $y_{v}$ denote the already stacked and statically weighted basis within the larger training matrix X, dilation Jacobian, and unknown vector for spectral band v. The adaptive robust weight is updated from the current per-spectral-band residual

$$r_{v}^{\left(k\right)}=\sqrt{w_{\mathrm{rob},v}^{\left(k\right)}}y_{v}-\sqrt{w_{\mathrm{rob},v}^{\left(k\right)}}\phi_{v}^{\left(k\right)}(B_{v}+\gamma_{v}^{\left(k\right)}J_{v})a^{\left(k\right)},$$ (57)

by first computing a robust scale

$$\sigma_{v}^{\left(k\right)}=1.4826\;\mathrm{MAD}(r_{v}^{\left(k\right)}),$$ (58)

then forming

w^rob,v(k+1)={1,σv(k)≤0,clip(δrobδrob+σv(k),10−3,1),σv(k)>0, (59)

and finally smoothing it as

wrob,v(k+1)=ηrobw^rob,v(k+1)+(1−ηrob)wrob,v(k). (60)

After updating the robust weight, the probability vector, primary, and dual variables are updated. Afterwards, the dilation correction term is updated, which uses the local Jacobian $J_{v}$, whose columns are finite-difference approximations of a normalized radial dilation operator;

$$J_{v,:,j}\approx \frac{D_{1+\varepsilon }(B_{v,:,j})-D_{1}\left(B_{v,:,j}\right)}{\varepsilon },$$ (61)

and updates $\gamma_{v}$ by a clipped Newton step. With

rv=y˜v−y^v, (62)

$${\overset{˜}{y}}_{v}=\sqrt{w_{\mathrm{rob},v}}y_{v},$$ (63)

y^v=wrob,vϕv(Bv+γvJv)a, (64)

and

$$q_{v}=\phi_{v}\sqrt{w_{\mathrm{rob},v}}J_{v}a,$$ (65)

the gradient and Hessian are

$$g_{\gamma ,v}=-q_{v}^{⊤}r_{v}+\lambda_{\gamma }\gamma_{v},$$ (66)

$$h_{\gamma ,v}=q_{v}^{⊤}q_{v}+\lambda_{\gamma },$$ (67)

so that

$$\gamma_{v}\leftarrow \text{clip}(\gamma_{v}-\alpha_{\gamma }\frac{g_{\gamma ,v}}{h_{\gamma ,v}},\gamma_{\min},\gamma_{\max}).$$ (68)

Finally, after $\gamma_{v}$ is updated, the per-band amplitude scale ϕ is obtained by minimizing

$$\frac{1}{2}\|{\overset{˜}{y}}_{v}-\phi_{v}m_{v}{\|}_{2}^{2}+\frac{\lambda_{\phi }}{2}(\phi_{v}-1{)}^{2},$$ (69)

$$m_{v}=\sqrt{w_{\mathrm{rob},v}}(B_{v}+\gamma_{v}J_{v})a,$$ (70)

which gives the closed-form update

$$\phi_{v}\leftarrow \text{clip}(\frac{m_{v}^{⊤}{\overset{˜}{y}}_{v}+\lambda_{\phi }}{m_{v}^{⊤}m_{v}+\lambda_{\phi }},\phi_{\min},\phi_{\max}).$$

Once all adaptive variables had been updated, iterations were terminated when the primal and dual residual tolerances were satisfied or when the maximum iteration count was reached.

## Data Availability

The data, materials, and custom analysis code supporting the findings of this study are available from the corresponding authors upon reasonable request, subject to institutional and ethical considerations.
